# Influence of Newborns’ Characteristics on Postpartum Depression: The Impact of Birth Season and Male Sex in a Romanian Cohort Study

**DOI:** 10.3390/diagnostics14212455

**Published:** 2024-11-01

**Authors:** Silvia Onuc, Gheorghe Mihail Banariu, Sergiu Ioachim Chirila, Cristian Delcea, Costin Niculescu, Mihaela Rus, Diana Badiu, Vlad Tica

**Affiliations:** 1 Obstetrics & Gynaecology Department, Faculty of Medicine, “Ovidius” University from Constanta, 900470 Constanța, Romania; silviaizvoranu@yahoo.com (S.O.); costinniculescu@yahoo.com (C.N.); vtica@eeirh.org (V.T.); 2 “Sf. Apostle Andrei” University Emergency County Hospital Constanta, 900591 Constanța, Romania; 3 Faculty of Medicine, “Ovidius” University from Constanta, 900470 Constanța, Romania; banariu.gheorghe@gmail.com (G.M.B.); sergiu.chirila@univ-ovidius.ro (S.I.C.); 4 Department of Forensic Medicine, “Iuliu Hațieganu” University of Medicine and Pharmacy, 400012 Cluj-Napoca, Romania; 5 Faculty of Law and Administrative Sciences, “Ovidius” University from Constanta, 900470 Constanța, Romania; psiholog_m@yahoo.com; 6 Romanian Academy of Scientists, 50044 Bucharest, Romania

**Keywords:** postpartum depression, birth, winter season, male sex, public health system

## Abstract

Background: Although risk factors associated with maternal postpartum depression (PPD) have been recognized, it is still unknown how some newborn characteristics could influence the appearance of PPD. Aim: Our research aimed to unravel the impact of a newborn’s features on women with PPD. Methods: The study was conducted in the Obstetrics and Gynecology Department at our University Emergency County Hospital, between August 2019 and April 2021. We included 904 women from the second day of the postpartum period, divided into two groups: women with PPD (*n* = 236) and control (i.e., women without PPD, *n* = 668), by using the Edinburg Postpartum Depression Scale. Characteristic information on the newborns (i.e., the months in which they were born, premature delivery, birth weight, or sex) was evaluated. Results: Our results suggest that the winter season (i.e., December and January months, *p* = 0.01) births and male newborns (*p* = 0.02) were strongly related with the appearance of depressive symptoms during the postpartum period. Conclusions: Therefore, our study sustains that women who give birth to male newborns in the winter season are more prone to developing PPD. This should be analyzed by all public health care systems in order to prevent such a condition earlier in certain groups of women.

## 1. Introduction

Postpartum depression (PPD) represents a serious condition with symptoms like decreased concentration and hopeless mood and is observed in 10% to 20% of all postpartum women [1,2,3]. Following symptom onset, PPD seems to have long-term negative consequences on both mothers and newborns, being an important issue in a suicidal state [4]. Unlike the temporal “baby blues”, PPD seems to manifest more severely and last for months, with the symptomatology of sadness, low self-esteem, guilty feelings, depressed mood, sleep and appetite disturbances, social communication disordering, self-harming, irritability and anxiety and scarceness, especially regarding newborn care. In addition, all the people in the mother’s life are affected [4].

Recognizing PPD in time is crucial for the health and well-being of both mother and their newborns [5]. However, up to 50% of PPD cases still remain undiagnosed due to women's hesitance, which involves stigma or blemish around PPD symptoms, behavior changes and fears of abandonment by their family or relatives [5].

The Edinburgh Postpartum Depression Scale (EPDS) is most often used in order to diagnose clinical PPD. In this way, the American College of Obstetricians and Gynecologists, the American Academy of Pediatrics, and the American Academy of Family Medicine recommend screening for PPD using the EPDS [6].

Several risk factors associated with PPD have already been studied, like biological factors (i.e., complications during pregnancy, and abortions in previous medical pathological history), as well as psychosocial factors (i.e., lower social support, psychosocial stressors, unwanted pregnancy, child abuse, families’ issues or lower level of education), including culture and traditions from each country [7]. Although the risk factors are presently well-known, less than half of women with PPD are nowadays diagnosed [8,9]. In the last few decades, it was seen that depression is mostly increasing in all countries, being the fourth leading cause of disease burden, causing different impairments. Moreover, considering the poor economic status in some countries, the level of the mother’s acknowledgement is still low regarding various health-related programs. Therefore, screening should be used in order to identify and prevent such causes. Various different professionals or therapies could be included in these programs to solve the consequent issues [8].

When a child is born, it represents a happy event for the entire family. But, also in that time, the mothers can present negative feelings and self-doubt postpartum, having a more depressive state. Most likely women do not show these feelings to other people, like family members, relatives or health professionals, which affects the child’s mental growth and development [10]. Another study shows that mothers who were depressed in the first 3 months of their newborn’s life were seen to be more irascible and less involved in the care of their newborn [11].

It was demonstrated that there is a connection between maternal PPD and breastfeeding, maternal–infant bonding, and care of the infant and other children, including the bonding with her partner. In addition, other features have consequences on newborns like birth season, poor nutrition or abnormal infant development, especially in terms of psychological development. Further, these can lead to poor planning for ongoing mental health development such as cognitive performance, causing hyperactivity and depression [12]. Interestingly, in other nomenclatures, the criteria for PPD are similar to major depressive disorder, which can occur a few weeks after birth [9].

Starting with pregnancy, women’s bodies achieve different adaptations in order to maintain proper fetal development. However, messages from the placenta to the fetus are important in sustaining its normal state [13]. Further, it seems that the placenta could influence the fetus’ sex and sustain its pathophysiological implication in pregnancy [14,15]. However, a male fetus has been shown to lead to chronic inflammation of the placenta with activated pro-inflammatory cytokines in the circulation [16,17]. Similar studies have attributed the role of male babies on maternal reproductive hormone levels by increasing inflammation [17,18]. Therefore, we suppose that further maternal depressive symptoms will be influenced by the fetus’ sex, with a higher tendency in male pregnancies. One study sustains this fact and showed that PPD had a higher percentage of developing in women carrying a male fetus along with other birth complications [19].

Besides male sex, the birth season is also shown to influence the appearance of PPD. To reinforce this hypothesis, some studies showed that more cases of women with PPD were seen in the winter season [20,21]. In contrast, Henriksson and contributors show that seasonal patterns do not directly influence depressive symptoms [22]. However, the authors showed that the seasonal patterns when correlated with newborn sex seem to depend on other factors related to specific climatic or geographic conditions [22]. Therefore, if fetus sex and seasonality can affect PPD, public health systems should take better preventive measures in order to detect the appearance of PPD in such groups of women.

Knowing how depressive symptoms influence a woman’s postpartum period according to a newborn’s characteristics (i.e., the months in which they were born or premature delivery, birth weight or sex), would also contribute to a better understanding of the appearance of PPD over time.

Therefore, the main purpose of this study is to show the impact of newborn characteristics in women with PPD.

## 2. Materials and Methods

### 2.1. Design and Sample

We conducted an observational prospective study on immediate postpartum women who delivered in the Obstetrics and Gynecology Department from “Sf. Apostle Andrei”, Emergency Clinic County Hospital of Constanta, South-East of Romania. A sample of 904 women at high risk of depression on the 2nd day of postpartum period was used, divided into 2 groups: women with PPD (*n* = 236) and control (i.e., women without PPD, *n* = 668), by using EPDS. The inclusion criteria for the women were: (1) women between 18 and 45 years old; (2) women who give birth to a single baby; (3) women without physical or mental abnormalities; (4) women without any additional treatment, including psychiatric disorder that required treatment; (5) women who accepted to participate voluntarily in the study; and (6) women who speak in the Romanian language.

### 2.2. Newborns Characteristics Information

Between August 2019 and April 2021, characteristic information on newborns from medical electronic records was assessed as follows: the months in which they were born, premature delivery (i.e., <37 gestational weeks), birth weight in grams (<2500 g vs. ≥2500 g), and newborn sex (male vs. female). Newborns were eligible to take part in the study if they were 2–3 days of age, and if they resided in the study area. Newborns with obvious, severe congenital abnormalities, hemoglobin <7 g/dL (normal values = 13.4–19.8 g/dL), and those who were receiving special nutritional supplements as part of a feeding program were excluded.

All participants were informed of the aims and procedures and had the option to withdraw from the study at any time. The study was conducted in accordance with the Helsinki Declaration on Human Rights and the informed consent from all participants in the study as well as the Agreement of the Ethics Commission (No. 29726/31.05.2021) were obtained.

### 2.3. Statistical Analysis

The statistical analysis was performed using the IBM SPSS statistics for Windows, Version 28.0 (Armonk, NY, USA: IBM Corp). The procedures used were descriptive statistics, graphs, and non-parametric statistical tests. Data are presented as mean, standard deviation, median, minimum and maximum, for continuous variables, or as percentages for categorical variables. For hypotheses testing, Independent Samples Mann–Whitney U test, Independent Samples T-test, Chi-square test of association, and Chi-squared test for the comparison of two proportions were used, depending on the type of analyzed variables. The significance level was set at 0.05.

## 3. Results

From the total number of patients (*n* = 904), the presence of PPD was only seen in 26.10% (*n* = 236). The rest of the patients (*n* = 668) were women without PPD (control group).

**Figure 1:**
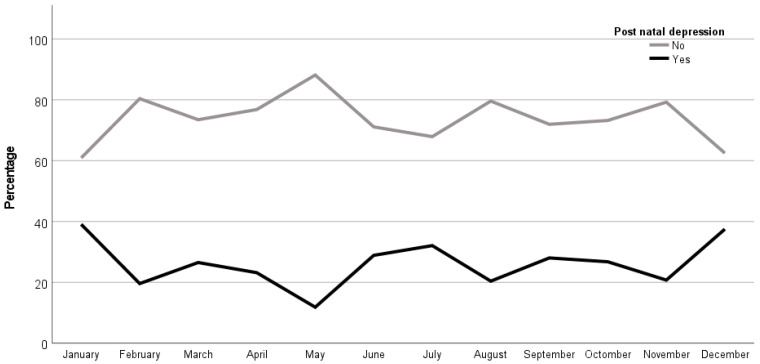
Distribution of newborns from women with and without PPD according to the months in which they were born.

By comparing the association between the month of birth and the presence of PPD, it was shown that there were higher values in newborns from women with PPD in the months of winter (i.e., December with 37.5%, *n* = 3, and January with 39.1%, *n* = 34) than the newborns from women without PPD in the summer months (i.e., May with *n* = 67, 88.2% and August with *n* = 78, 79.6%), all these being statistically significant (*p* = 0.018). Therefore, the births within the winter season led to a higher proportion of women with PPD compared to women without PPD in which the majority of them gave birth in the summer season (Figure 1). Therefore, the winter season, and in particular, the months of December and January, could represent a potential risk factor for the appearance of PPD.

Comparing the two groups of newborns, it was shown that there was no statistically significant association of premature birth (i.e., <37 gestational weeks) between women with PPD and without PPD (*p* = 0.654). From the group of women with PPD (*n* = 236, 26.1%), 16 (6.8%) newborns were premature (i.e., <37 gestational weeks), in comparison with the group of women without PPD (*n* = 668, 73.9%), in which 40 (6.0%) newborns were born prematurely (*p* = 0.654).

**Figure 2:**
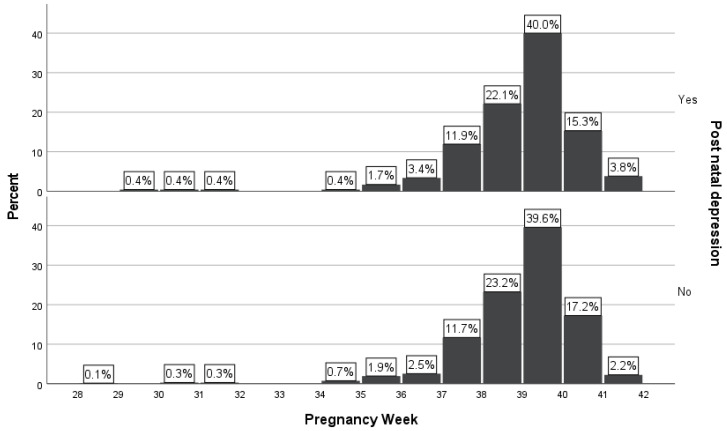
Distribution of newborns from mothers with and without PPD according to prematurity.

The number of newborns from women with PPD (*n* = 219, 93.2%) born at ≥37 gestational weeks was similar to newborns from women without PPD (*n* = 628, 94.0%) born at ≥37 gestational weeks (Table 1 and Figure 2). Both women with and without PPD showed the same tendency to give birth after 37 gestational weeks.

**Figure 3:**
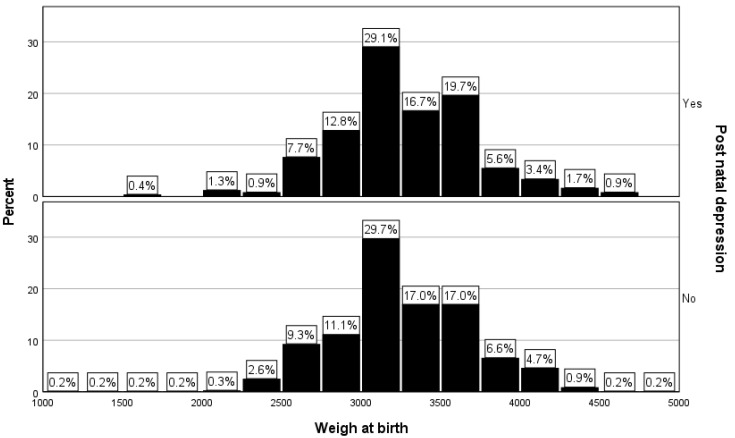
Distribution of newborns from women with and without PPD according to birth weight.

The percentage of low-birth-weight newborns was 2.6% (*n* = 6) in the PPD group, while for the women without PPD, the percentage of low-birth-weight newborns was 3.4% (*n* = 23). The observed difference was still not statistically significant between the two groups (Table 1 and Figure 3, *p* = 0.51).

**Figure 4:**
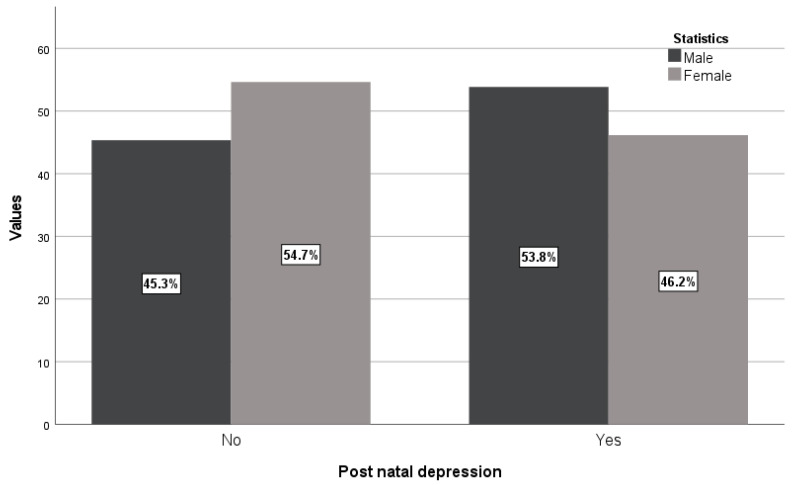
Distribution of newborns from women with and without PPD according to newborn sex.

There were more male newborns compared with female newborns from women with PPD (53.8% vs. 45.3%) in contrast with values of newborns from women without PPD (46.2% vs. 54.7%) with a statistically significant association (Table 1 and Figure 4, *p* = 0.025). These results show that women with PPD give birth to more male newborns and women without PPD give birth to more female newborns, meaning male newborns could represent a possible risk factor for PPD appearance.

## 4. Discussion

A quantitative cross-sectional study conducted in Jordan on 188 mothers using EPDS showed that infant characteristics such as gestational age, medical condition classification, and birth weight are found to be significant factors that influence PPD appearance among mothers [23].

Infants raised by depressed mothers show confusion in social and emotional environments, including low attachment to their mothers, as well as greater vulnerability to anxiety disorders [12].

In the present study, from all newborn characteristics such as the months in which they were born, premature delivery, birth weight and newborn sex, only the months of birth (i.e., December and January months) and the newborn’s sex being male were statistically significant in women with PPD compared to women without PPD. Our results are in accordance with another study conducted on 298 postpartum women from the United Kingdom who found that the rate of PPD went up to 71–79% among women giving birth to male infants, along with other birth complications [19].

Another study found that 181 women with PPD were associated with having a newborn that was of the male gender, significantly reducing their quality of life [24]. Another study achieved from Sweden showed that women had a higher risk of PPD 5 days after the delivery of a male fetus [25]. The study by Hofheimer and contributors [26] found higher PPD rates associated with anxiety, prenatal marijuana use, or male infants. Therefore, other factors like being a single woman, or having low education associated with PPD were not seen as significant in this study, which also included newborn medical complications.

Interestingly, the associations of PPD with socioemotional newborn development in the study of Subbiah and contributors [27] emphasizes the need for effective and regular screening of PPD during the postpartum period. This study shows that PPD had a negative impact on newborn socioemotional evolution and that maternal self-efficacy could have a potential role in such conditions [27]. However, mothers with PPD, together with their newborns, could benefit from healthcare facilities and should focus on preventing the appearance of PPD.

Some physiological mechanisms like sex-differential shifts and biomarkers from the maternal reproductive system reinforce our statements. Estradiol, the hormone that blends the serotonergic system together with progesterone levels decreases [28]. The pathophysiology of PPD can be caused by alterations in the immunological and endocrine systems, in which the hypothalamic–pituitary–adrenal axis and lactogenic hormones are noted [5]. This hypothalamic–pituitary–adrenal axis produces the release of cortisol, especially in stress conditions. When the hypothalamic–pituitary–adrenal axis is affected, the release of catecholamines is much lower. Therefore, in pregnancy, the hypothalamic–pituitary–adrenal axis is increased and remains in this condition for up to 12 weeks postpartum [5].

However, the different association between newborn sex and PPD is still under debate. It was shown that altered self-reported autonomic reactivity was associated with a worse mental health population. One study assesses the impact of 170 women (i.e., in the second or third trimester and one month after delivery) prepartum self-reported autonomic reactivity on the development of postpartum depressive symptoms. By using the Body Perception Questionnaire—Short Form, the authors evaluated the autonomic functions related to the organs above and below the diaphragm. The results showed that the evaluation of self-reported autonomic activity may be an important instrument to antenatally identify women at risk of PPD condition [29]. 

The present study represents a continuation of our previous research in which we emphasise the significant risk factors for PPD among women from South-East Romania, like the women’s age and the level of education [30]. Therefore, screening for PPD in such women could lead to a decrease in depressive symptoms [31,32]. Seasonal mechanisms like the winter season causing the appearance of PPD are still not well understood. When the women are exposed to higher intensity light, (i.e., during the summer season), it was shown to have a positive impact on their mental health, in contrast to the winter season, when the day becomes much shorter with a lower intensity and could present a negative impact [33,34]. Blume and contributors also showed the same results in which reduced day length and less exposure to light may be correlated with a depressive disposition [35]. Moreover, engagement of such women in different activities appeared to be connected with positive self-esteem [36], which can reduce anxiety and PPD appearance.

Promoting both support and therapies for these women, especially by raising a connection with other people during the winter season could reduce the symptomatology of the PPD condition [37]. A protective role was seen when such support came much earlier in high-risk postpartum women [38].

Fluctuating family conditions, which can influence depressed mothers’ children’s characteristics, are represented by the sample heterogeneity and mainly risk factors like low social support and financial stress. Interestingly, stressful factors for mothers can contribute to the appearance of different issues in newborns [39]. All these factors are constructed on the complexity of the relationship between the maternal condition and newborn appearance, including mother–newborn interactions and birth predisposition.

The results of the study by Romeo et al. [40] showed that the emotions section associated a significant bond between the fear of childbirth and cesarean section predisposition (*p* < 0.0001). Specific fears included the pain of contractions, the inability to manage the event, adverse outcomes for the fetus and the duration of labor. We believe that these fears could be extended also in the postpartum period and have a role in PPD enhancement.

Considering these risk factors, public health systems should pay more attention to new mothers who are at high risk for PPD providing prompt interventions and treatment, especially in the early stages [41]. Furthermore, these factors can be included in hospital policies or preconception programs to make all mothers who attend such a programme aware of PPD.

Education and information are essential components in addressing PPD, especially in the early stages. The attitude about such risk should begin even in prenatal care, and professionals should inform expectant mothers and their families about the potentially positive signs and symptoms [5]. Afolabi and contributors [42] observed that wealth status was a significant predictor of unintended pregnancy among older married women. Moreover, fewer resources and less education may mean the mother faces borders in accessing quality healthcare programmes. Interestingly, the study of Navabinejad et al. [43] identified an intense emotional response to miscarriage, including grief, loss, guilt, and anxiety, which looked similar to the postpartum period.

Depression is usually medically healed, and depressed mothers can benefit from both pharmacological and support-based resources such as ‘talking’ and open discussions about such issues, giving way to the opportunity for intervention and therapies [44]. Using EPDS, Rizzo and contributors [45] showed that only 4.5% of a 110-person sample developed PPD at 3 or 6 months. They showed that agoraphobia, depressed mood, social anxiety and eating problems had a major impact on PPD appearance. Moreover, the study of Risso and Marra [46] sustained the fact that the relationship between temperament, character, and organizational well-being could have deeper implications in developing the mood or social behavior in each individual’s character, especially in special conditions.

The limitation of our study consists of potentially restricted risk factors of the newborns analyzed, which could have also included the type of birth (caesarean section versus vaginal births), Apgar score, breastfeeding, or the monitoring of PPD appearance in the dynamics of each pregnancy trimester.

However, the association between birth season, male fetal sex and PPD could help to explore the cause of PPD more deeply, thus preventing the occurrence of PPD in particular groups of women.

## 5. Conclusions

Professionals should make overall decisions in PPD prevention, in which families can be easily identified and receive immediate adequate support. Finally, risk factors for PPD such as male infants and winter seasonal births should be considered separately by all health public systems including hospitals, and based on this, such mothers should receive more attention in terms of possible PPD appearance.

## Figures and Tables

**Table 1:** Comparisons of characteristics between newborn groups from women with and without PPD.

Basic Characteristics	Newborn from Mothers with PPD * [233, N (%)]	Newborn from Mothers	*p*-Value Without PPD * [668, N (%)]
Premature delivery (<37 gestational weeks)	16 (6.8)	40 (6)	0.654
Low birth weight (<2500 g)	6 (2.6)	23 (3.4)	0.510
Male sex	126 (53.8)	302 (45.3)	0.025

* PPD = postpartum depression.

## Data Availability

The raw data supporting the conclusions of this article will be made available by the authors upon request.
